# Headaches, mental health disorders, and religiosity: analysis of the Saudi National Mental Health Survey

**DOI:** 10.3389/fpubh.2026.1813677

**Published:** 2026-04-15

**Authors:** Sameer Desai, Lisa Bilal, Abdulhameed Alhabeeb, Abdullah Alsubaie, Yasmin Altwaijri

**Affiliations:** 1 Innovation and Research, King Faisal Specialist Hospital & Research Centre, Riyadh, Saudi Arabia; 2 National Center for Mental Health Promotion, Ministry of Health, Riyadh, Saudi Arabia; 3 Department of Psychiatry, Edrak Medical Center, Riyadh, Saudi Arabia

**Keywords:** Arabs, cross-sectional, epidemiology, headache, mental health, migraine, Muslim, religion

## Abstract

**Background:**

Headaches have been consistently associated with mental health disorders. However, current prevalence of headaches in Arab populations is highly varied. Additionally, the potential role of religiosity in this relationship has not been studied. This study aimed to estimate the prevalence of headaches in Saudi Arabia, a highly religious Arab society, to examine their association with mental health disorders, and to explore whether religiosity modifies this relationship.

**Methods:**

This study used the Saudi National Mental Health Survey (SNMHS), a nationally representative, cross-sectional, community-based psychiatric epidemiological household survey. Trained interviewers assessed history of headaches, and common DSM-IV mental health disorders were diagnosed using the Composite International Diagnostic Interview (CIDI). Religiosity was measured using a validated culturally appropriate religiosity scale embedded within the survey. Headache prevalence was calculated as the proportion of respondents reporting headaches relative to the total sample. Survey-weighted logistic regression models were used to estimate adjusted association between headaches and mental health disorders. An interaction term was introduced to explore the role of religiosity.

**Results:**

The overall lifetime prevalence of headaches among Saudis was 56% (95% *CI*: 53%−58%), with 28% reporting recent episode of headaches. In multivariable logistic regression models including sociodemographic factors, respondents with headaches were more likely to have mental health disorders (*OR*: 2.09, 95% *CI*: 1.50–2.91; *p* < 0.001). Religiosity did not modify the association between headaches and mental health disorders (interaction *OR*: 1.00, 95% *CI*: 0.98–1.02; *p* > 0.9).

**Conclusion:**

Headaches are highly prevalent in Saudi Arabia, with more than half of respondents reporting a lifetime history and nearly one-third experiencing recent episodes. Individuals with headaches are more likely to have mental health disorders. However, varying levels of religiosity do not appear to modify this relationship. These findings underscore the importance of screening for mental health disorders in individuals presenting with headaches, regardless of their religiosity, and highlight the potential value of collaborative models that integrate professional mental health support with religiously sensitive approaches.

## Introduction

Mental health disorders are among the leading causes of global health-related burden, affecting over 1 billion people worldwide (1). Their prevalence has increased over the past three decades and is expected to continue rising (2). While economic consequences as a result of mental health disorders are also significant with billions of dollars lost annually due to work productivity losses (1). Together, these data highlight a global mental health crisis that warrants immediate public health attention.

A condition that has been consistently linked with mental health disorders is headaches. Epidemiologic studies estimate that headaches, similar to mental health disorders, are also highly prevalent and estimated to occur in approximately 52% of the population (2). Many studies report higher prevalence of mental health disorders among individuals experiencing headaches (3–6). Headaches are commonly classified into two main types, tension-type and migraines. Tension-type headaches are typically described as bilateral, pressing or tightening in quality, of mild-to-moderate intensity, without associated nausea, and not aggravated by routine physical activity (7). Migraines are recurrent headaches that are often unilateral, pulsating in nature, and of moderate to severe intensity, usually accompanied by nausea as well as sensitivity to light and sound (7). While both headache types have been associated with mental health disorders, evidence suggests that migraines demonstrate a stronger association (4, 8, 9). The direction of this relationship, however, remains uncertain. Evidence from multiple longitudinal and cohort studies supports a bidirectional association (10). Headaches increase the risk of subsequent mental health disorders, while pre-existing mental health disorders predict the onset of new headaches (10). Shared biological and psychosocial mechanisms have been proposed to underlie this potential bidirectional link (11, 12). Regardless of direction of the relationship, the link between headaches and mental health disorders is well established (3, 4, 8, 10, 13, 14).

One aspect of the relationship between headaches and mental health that has not been studied is the role of religion, although over 80% of the world's population is religiously affiliated (15). The consensus in the literature, largely derived from Western, high income countries, is that individuals with stronger religious and spiritual engagement tend to report enhanced well-being, quality of life, and overall life satisfaction (16–21). There is also substantial evidence suggesting that engagement in religious activities may have a protective effect against the development of certain mental health disorders (22, 23). However, despite the extensive literature highlighting the positive association of religion and spirituality on mental health, their specific role among individuals with headaches and mental health disorders has not been fully investigated. Given the centrality of religion and the relatively young demographic profile of Saudi Arabia, this setting provides a strategic context in which to examine the potential moderating role of religion in mental health outcomes within a non-Western context.

The objective of the present study is to estimate the prevalence of headaches, second, to confirm the association between headaches and mental health disorders in a non-Western society, and lastly, to explicitly explore the role of religion among individuals experiencing headaches and mental health disorders, using a nationally representative survey in Saudi Arabia.

## Methods

### Data source

The Saudi National Mental Health Survey (SNMHS) was used for this study. It is the first nationally representative, cross-sectional, community-based psychiatric epidemiological household survey conducted in the Kingdom of Saudi Arabia between 2014 and 2016. The survey was a joint effort between King Salman Center for Disability Research, King Faisal Specialist Hospital and Research Centre, the Saudi Ministry of Health, King Saud University, the Ministry of Economy and Planning, and the General Authority for Statistics. The survey targeted Saudi citizens aged 15–65 years living in most administrative regions of the Kingdom. Citizens from Jazan and Najran (representing ~8% of the population) were excluded due to security concerns at the Saudi–Yemeni border at the time of the survey. Individuals residing in institutions were also not included.

The SNMHS was implemented as part of the World Health Organization (WHO) World Mental Health (WMH) Initiative, the largest coordinated series of cross-national psychiatric epidemiological surveys ever conducted. These epidemiologic surveys employ rigorous procedures and standardized protocols (24). The SNMHS, like other WMH surveys, was conducted in two parts to reduce respondent burden. The first part, administered to all participants, collected standard demographic and socioeconomic information as well as core mental health disorders. The second part was administered only to respondents who met lifetime criteria for at least one mental health disorder (cases) and to a 25% probability subsample of respondents who did not meet these criteria (controls). This section assessed additional mental health disorders of secondary interest, as well as treatment history, healthcare utilization, and other correlates. The design of the survey includes probability-based stratified multi-stage clustered area probability sampling and was administered through face-to-face interviews. Detailed descriptions of survey design, sampling strategy, and interviewing procedures are published elsewhere (25–27). Study procedures conformed to the international standards set by the Declaration of Helsinki. Written informed consent was obtained from respondents prior to beginning each interview. These consent procedures were approved by the Institutional Review Board at the King Faisal Hospital and Research Center (RAC# 2091093).

### Analytic sample

A total of 4,004 Saudi residents participated in the national survey, with a response rate of 61%. For the current study, descriptive analyses were based on the full sample (*N* = 4,004) and inferential analyses were based on the Part 2 subsample (*n* = 1,981), which included assessment of all mental health disorders as well as the in-depth religiosity questions relevant to the study objectives. For objective 3, a total of 107 (5.4%) respondents were excluded for missing data related to the religiosity questions.

### Measures

#### Mental health disorders

Mental health diagnoses were assessed using the WHO Composite International Diagnostic Interview (CIDI) Version 3.0, the same structured diagnostic tool employed across all WMH surveys (28). For select disorders, recalibrations were applied to account for potential underdiagnosis in the Saudi population (29). The CIDI is a fully structured interview administered by trained lay interviewers that generates both ICD-10 and DSM-IV diagnoses. In the SNMHS, 19 disorders were assessed, including anxiety disorders (panic disorder, agoraphobia without panic, social phobia, generalized anxiety disorder, post-traumatic stress disorder, obsessive–compulsive disorder, separation anxiety disorder), mood disorders (major depressive disorder, bipolar I/II disorder), eating disorders (anorexia nervosa, bulimia nervosa, binge-eating disorder), disruptive behavior disorders (attention-deficit/hyperactivity disorder, conduct disorder, oppositional-defiant disorder, intermittent explosive disorder), and substance use disorders (alcohol and drug abuse/dependence).

For the current study, individuals meeting life-time criteria for any of the above-mentioned disorders were classified as having a mental health disorder. Headaches were assessed in the SNMHS using the question: “Have you ever had headaches?”, referring to any occurrence over the respondent's lifetime. This measure therefore reflects the lifetime prevalence of headaches. Respondents who reported a history of headaches were further asked, “Did you still have headaches or receive any treatment for them at any time during the past 12 months?” This question captures more current or active headaches. Additionally, those reporting headaches in the past 12 months were asked to classify their headache type as either tension-type (described to respondent as “usually occurs on both sides of your head, the pain is steady and is like a tight band around your head”) or migraine (described to respondent as “generally comes on suddenly like an attack, tends to be pounding, throbbing, or pulsating, may occur on one side of your head, is often accompanied by nausea or vomiting, and gets worse with physical activity”) based on ICHD-II criteria (7).

The SNMHS included the Islamic Religiosity Scale developed by Marwa et al. (30, 31). This validated 40-item scale comprehensively evaluates four domains of Islamic religiosity: Worshiping, Islamic interests, Islamic behaviors, and Beliefs. The first three domains are measured on a 5-point Likert-type scale with response options ranging from “always”, “mostly”, “occasionally”, and “rarely” to “never”. Beliefs are measured on a strength scale (“very strong” “strong”, “middle”, “weak”, and “nothing”). Item responses were summed to produce a total raw score for each respondent. Higher scores indicated greater levels of Islamic religiosity, whereas lower scores indicated lower levels of religiosity. As per the scoring manual, summed raw total scores were categorized into four groups: No religiosity (< 50), Low religiosity (51–100), Medium religiosity (101–150), and High religiosity (151–200). Domain-specific scores were also calculated by averaging the item responses within each domain. The complete list of items included in the scale is provided in the Supplementary Figure S1.

The SNMHS also collected sociodemographic information, including gender (male, female), age at interview, education (low: 0–6 years; low average: 7–9 years; high average: 10–15 years; high: ≥16 years), marital status (never married, married, previously married), employment status (working, student, homemaker, retired, other), and household income (low, low average, high average, high).

#### Analysis plan

Descriptive statistics (means, medians, proportions, and frequencies) were calculated to summarize study sample characteristics. The prevalence of any life-time headache was calculated as the proportion of respondents who reported a life-time history of headache, divided by the total sample. Survey-weighted logistic regression models were used to estimate both unadjusted and adjusted associations between having headaches and the life-time prevalence of any mental health disorder, including socio-demographic characteristics that are known to affect health status. For the third objective, an interaction term between religiosity score (as a continuous term) and having headaches was included to examine whether the association varied by level of religiosity. Religiosity score was explored as a non-linear and categorical term. Stratified analyses by gender, headache type and separate analyses for specific mental health disorders were also explored. Analyses were conducted in R (version 4.3.0) (32) and incorporated sampling weights to account for differences between the study sample and the sociodemographic and geographic distribution of the census population of Saudi Arabia, as well as differential probabilities of selection within households and non-response bias (27). Weighting was also applied to data from Part II of the survey to account for the under-sampling of Part I respondents without core disorders using the R package *svyglm*. Appropriate statistical tests for weighted statistics were also used. Statistical significance was defined as *p* < 0.05 for all two-tailed tests.

## Results

### Socio-demographic characteristics

Table 1 presents the weighted characteristics of the analytic sample. The sample was evenly distributed between males and females, with a median age of 33 years (*SD*: 13). Nearly two-thirds of respondents were students or employed, while approximately one-quarter were homemakers. Half of the sample was married, and 43% had never been married. For education, 68% had attained high or above-average educational levels (10+ years; high-school or more). Forty-three percent of the sample was considered to have “low” income.

**Table 1:** Unweighted frequencies and weighted percentages of the overall study sample by headache status, Saudi National Mental Health Survey (*n* = 4,004).

Characteristic	Overall sample	By Headache status		
	*N* = 4,004^a^	No, *N* = 1,767	Yes, *N* = 2,237	*P*-value^b^
Characteristic				
Sex				
Female	2,105 (49%)	701 (37%)	1,404 (59%)	< 0.001
Male	1,899 (51%)	1,090 (63%)	809 (41%)	
Age, years	33 (13)	33 (13)	32 (13)	0.088
Marital status				
Never married	1,313 (43%)	591 (43%)	722 (43%)	>0.9
Married	2,454 (51%)	1,104 (51%)	1,350 (51%)	
Separated/divorced/widowed	237 (6.2%)	96 (6.1%)	141 (6.3%)	
Employment status				
Working	872 (26%)	357 (24%)	515 (27%)	< 0.001
Student	1,454 (37%)	748 (42%)	706 (33%)	
Homemaker	1,122 (22%)	398 (17%)	724 (27%)	
Retired	201 (5.3%)	115 (6.2%)	86 (4.5%)	
Other	355 (9.7%)	173 (11%)	182 (9.0%)	
Income category				
Low	1,494 (39%)	611 (37%)	883 (41%)	0.3
Low average	440 (11%)	206 (11%)	234 (11%)	
High average	644 (17%)	292 (17%)	352 (18%)	
High	1,426 (32%)	682 (35%)	744 (30%)	
Education category				
Low	741 (17%)	335 (17%)	406 (16%)	0.5
Low average	571 (15%)	274 (16%)	297 (14%)	
High average	1,923 (50%)	853 (50%)	1,070 (50%)	
High	769 (18%)	329 (17%)	440 (20%)	

^a^*n* (unweighted) (%); mean (*SD*);

^b^chi-squared test with Rao and Scott's second-order correction; Wilcoxon rank-sum test for complex survey samples.

Table 2 presents details on headaches. The overall lifetime prevalence of headaches among Saudis was 56% (95% *CI*: 53–58%), with 28% of the respondents reporting having current headaches. The median age of headache onset was 19 years (*SD*: 9). Tension-type headaches were more common (57%) than migraine-type headaches (44%).

**Table 2:** Unweighted frequencies and weighted percentages of headache information, Saudi National Mental Health Survey (*n* = 4,004).

Variable	*N* = 4,004^a^	95% *CI*^b^
Headaches, lifetime	2,213 (56%)	53%, 58%
Headaches, past 12 months	1,156 (28%)	26%, 30%
Age of onset of headaches, years (*n* = 1,782)	19 (9)	–
Migraine type headache (*n* = 790)	342 (44%)	39%, 49%
Tension type headache (*n* = 811)	421 (57%)	52%, 62%

^a^*n* (unweighted) (%); mean (*SD*);

^b^CI, confidence interval.

### Headaches and mental health disorders

Table 3 presents the unadjusted and adjusted weighted logistic regression models with odds ratios (ORs). In the unadjusted model, those who had headaches were more likely to suffer from mental health disorders (*OR*: 1.97, 95% *CI*: 1.39–2.71, *p* < 0.001) compared with those without headaches. In the multivariable model that included sociodemographic variables, the relationship between headaches and mental health disorders persisted and became slightly stronger (*OR*: 2.09, 95% *CI*: 1.50–2.91, *p* < 0.001). When examining individual mental health disorders, agoraphobia, social phobia, post-traumatic stress disorder, adult separation anxiety disorder, obsessive-compulsive disorder, mood disorders, conduct disorder, and binge-eating disorder were all significantly associated with headaches (Supplementary Table S1). Gender did not modify the relationship between headaches and mental health disorders (Supplementary Table S2).

**Table 3:** Unadjusted and adjusted weighted logistic regression results (odds ratios) for relationship between having headaches and life-time prevalence of mental health disorders of subsample (*n* = 1,981).

Variable	Unadjusted			Adjusted		
	*OR* ^a^	95% *CI*^a^	*p*-value	*OR* ^a^	95% *CI*^a^	*p*-value
Headaches (ref: no)	1.94	1.39, 2.71	< 0.001	2.09	1.50, 2.91	< 0.001
Sex (ref: no)						
Female				0.98	0.65, 1.49	>0.9
Age, years (per 1-year increase)				0.96	0.94, 0.98	< 0.001
Marital status (ref: never married)						
Married				1.39	0.84, 2.30	0.2
Separated/divorced/widowed				3.15	1.48, 6.72	0.003
Employment status (ref: working)						
Student				0.82	0.45, 1.49	0.5
Homemaker				0.85	0.49, 1.47	0.6
Retired				1.04	0.40, 2.70	>0.9
Other				1.03	0.58, 1.85	>0.9
Income category (ref: low)						
Low average				0.96	0.51, 1.83	>0.9
High average				1.13	0.71, 1.80	0.6
High				1.07	0.72, 1.58	0.8
Education category (ref: low)						
Low average				2.12	1.15, 3.90	0.016
High average				1.79	1.07, 3.02	0.028
High				1.56	0.87, 2.82	0.14

^a^OR, odds ratio; CI, confidence interval.

### Religiosity and mental health disorders

Table 4 reports the religiosity scores based on the Islamic Religiosity Scale. The median score was 179 (*IQR*: 166–189), and 92% of respondents were categorized as having “High religiosity”. Very few respondents exhibited no or low religiosity (*n* = 4). Table 5 presents the relationship between religiosity scores and life-time prevalence of CIDI/DSM-IV mental health disorders. In the unadjusted model, respondents with higher religiosity scores were less likely to have a mental health disorder (*OR*: 0.93, 95% *CI*: 0.88–0.97, *p* = 0.002) compared with those with lower religiosity scores. After adjusting for other covariates, the association slightly weakened (*OR*: 0.95, 95% *CI*: 0.90–1.00, *p* = 0.067) but remained positively associated. It is important to note that there was some imprecision in the adjusted odds ratio for this relationship, as reflected by the relatively wide confidence intervals. However, majority of the confidence interval distribution suggests that beneficial effects of higher religiosity on mental health disorders are more compatible with the data than no effect. Religiosity score was additionally evaluated as a non-linear and categorical variable, and the linear specification demonstrated the best model fit according to model fit statistics (Supplementary Tables S3–S5).

**Table 4:** Weighted religiosity scores of the study sample (*n* = 1,874).

Variable	*N* = 1,874^a^
Total religiosity score	179 (166, 189)
Religiosity level group	
High	1,720 (92%)
Medium	151 (8.3%)
Low	4 (0.1%)
Religiosity domains	
Beliefs	5.00 (4.67, 5.00)
Worshiping	4.33 (3.89, 4.56)
Interests	4.35 (3.82, 4.82)
Behaviors	4.88 (4.50, 5.00)

Scores range from 0 to 200, with higher values indicating greater levels of religiosity.

^a^Median (IQR); *n* (%).

**Table 5:** Unadjusted and adjusted weighted logistic regression results (odds ratios) for relationship between religiosity level (as continuous score) and mental health disorders.

Variable	Unadjusted			Adjusted		
	*OR* ^a^	95% *CI*^a^	*p*-value	*OR* ^a^	95% *CI*^a^	*p*-value
Religiosity level score (per 5-point increase)	0.93	0.88, 0.97	0.002	0.95	0.90, 1.00	0.067
Sex (ref: male)						
Female				1.17	0.77, 1.78	0.5
Age, years (per 1-year increase)				0.96	0.94, 0.99	0.003
Marital status (ref: never married)						
Married				1.46	0.86, 2.47	0.2
Separated/divorced/widowed				3.02	1.36, 6.71	0.007
Employment status (ref: working)						
Student				0.82	0.45, 1.51	0.5
Homemaker				0.87	0.49, 1.56	0.6
Retired				1.38	0.52, 3.67	0.5
Other				1.09	0.61, 1.96	0.8
Income category (ref: low)						
Low average				0.83	0.44, 1.55	0.6
High average				1.05	0.64, 1.71	0.8
High				1.06	0.71, 1.58	0.8
Education category (ref: low)						
Low average				1.97	1.07, 3.88	0.029
High average				1.93	1.10, 3.38	0.021
High				1.56	0.82, 2.97	0.2

^a^OR, odds ratio; CI, confidence interval.

### Religiosity, headaches, and mental health disorders

To explore whether the relationship between headaches and mental health disorders varied by religiosity levels, an interaction term between religiosity score and headaches was included in the model. The interaction term was not statistically significant (*OR*: 1.00, 95% *CI*: 0.98–1.02, *p* > 0.9; Table 6), indicating that the observed association between headaches and mental health disorders did not statistically differ across levels of religiosity. To better illustrate this relationship, Supplementary Table S6 and Supplementary Figure S2 present the predicted probabilities of having a mental health disorder from the regression model across different levels of religiosity, stratified by headache status. As expected, the probability of mental health disorders decreases with higher religiosity. However, the difference in predicted probability for having mental health disorders between respondents with and without headaches remains consistent at each level of religiosity, indicating that in our sample, religiosity does not modify the relationship between headaches and mental health disorders.

**Table 6:** Logistic regression results (odds ratios) for relationship between headache and mental health disorders, interacted with religiosity score.

Variable	*OR* ^a^	95% *CI*^a^	*p*-value
Headaches (ref: no)	1.72	0.05, 59.4	0.8
Religiosity level score (per 5-point increase)	0.98	0.97, 1.00	0.044
Headache × religiosity (interaction)^b^	1.00	0.98, 1.02	>0.9

^a^OR, odds ratio; CI, confidence interval,

^b^interaction term between headache status and religiosity score.

## Discussion

In this large nationally representative survey sample from a non-Western religious society, headaches were highly prevalent and were associated with mental health disorders, in line with prior literature. It was also found that, while higher levels of religiosity were generally associated with less mental health disorders, the association between headaches and mental health disorders did not vary by religiosity levels.

Headaches have received considerable attention in recent years due to their high global prevalence in all types of populations. In a recent review of 357 population-based studies published up to 2020, Stovner et al. estimated the global prevalence of active headache disorder (defined as occurrence within the past 12 months) to be 52%, with North African and Middle Eastern countries showing an estimated prevalence of 61% (33). In comparison, our study found a much lower 12-month headache prevalence of 28% and a life-time prevalence of 56%.

Our study's estimated prevalence of headaches is also markedly lower than findings from two recent Saudi-specific cross-sectional surveys (34, 35). Jumah et al. reported a 1-year headache prevalence of 66% (34), while Muayquil et al. (35) estimated 84%. However, the differences in prevalence estimates may be explained by major methodological issues. For instance, Muayquil et al. specifically sampled people from a large urban University in Riyadh (capital city of Saudi Arabia) which is not representative of the general Saudi population. This highly selective sample may explain the higher prevalence of headaches reported in their study. Jumah et al. surveyed nearly 2,000 individuals (including expatriates) across the Kingdom using random digit dialing of mobile phone numbers. This approach is known to underrepresent females and older adults who may have less access to mobile phones. Although statistical adjustments were made to account for these sex and age differences in their sample, Jumah et al. still reported doubling of the 12-month prevalence of headaches compared with the present study (66% vs. 28%).

Our study employed rigorous procedures such as random selection of household listings, face-to-face interviews, call-backs to ensure a representative or closely representative sample that was additionally corrected with sampling weighting methods. Our study's estimates were closer to Global Burden of Disease (GBD) estimates, which report a global headache prevalence of 36% and a Saudi-specific prevalence of 37.5% (36). GBD methods are well-established and adjust for multiple biases before estimating prevalences, thus not surprising their estimate is closer to our study.

Our study confirmed that headaches are associated with mental health disorders, a finding that has been well-established since the early 2000s but now also confirmed in a non-Western population. Radat and Swendsen (4) reviewed the literature and concluded that migraine (including tension-type headaches) is comorbid with a range of psychiatric conditions, particularly depression and anxiety. This association was further confirmed by Antonaci et al. (6), who reported that population-based studies consistently show an increased risk of affective and anxiety disorders among patients with migraine, with no major differences between tension-type and migraine headaches. Antonaci et al. also included a meta-analysis of 12 papers in their study and found that the odds ratios of depression for people who have migraine ranged from 1.8 and a maximum of 4.4 and the overall combined odds ratio was 2.2 (95% CI: 2.0 to 2.3). This estimate was similar to our estimate of 2.1 and means that the association is consistent with prior studies on similar topics. While initially studied in adults only, more recent research has demonstrated similar relationships in younger populations. For example, Orr et al. (3) repeated these findings among adolescents, and Hommer et al. (8) reported a similar association in American youth (*OR*: 2.74, 95% *CI*: 1.94–3.83), particularly with anxiety and depression. In our study, results were consistent with prior population-based literature, showing a two-fold increase in mental health disorders among individuals who reported headaches. Although headache subtype data were collected as part of the SNMHS, substantial missing responses limited their utility, and as such subtype-specific analyses were not conducted. Taken together, these findings reinforce the evidence that patients regardless of age, who present with headaches should be screened for co-occurring mental health disorders. Early identification of mental health disorders may have important implications for treatment and management. It also underscores the importance of now conducting high-quality prospective research on this topic that can inform our understanding of the risk-factors headaches or mental health that may serve as targets for prevention.

Religion has been a central aspect of human societies for millennia, shaping culture, social structures, and health behaviors around the world. Despite this, its relevance to mental health has often been overlooked within public health communities, although religion is practiced worldwide and can reasonably be considered a major social determinant of health (23). This neglect may stem from the fact that much of global mental health policy-making has been driven by largely secular countries, where religion is less prominent in public life (37). Nevertheless, the evidence linking religion and mental health is compelling and warrants greater attention. VanderWeele and Ouyang (23) reviewed the extensive literature on this topic and, focusing on methodologically rigorous studies (e.g., longitudinal, quasi-experimental, and those with strong confounding control), concluded that there is strong causal evidence supporting a beneficial effect of religious participation on mental health. Recently, Aggarwal et al. (22) systematically reviewed studies on religiosity and spirituality in young people and also concluded that interventions incorporating religious or spiritual practices were generally effective in reducing depression and anxiety. VanderWeele et al., argued that policymakers should consider integrating religious and spiritual dimensions into clinical and public health practice in an effort to improve the well-people of patients in light of this strong evidence. However, one limitation of the existing evidence is that it is drawn largely from Western, Christian-majority, and relatively secular populations, leaving questions about its generalizability to non-Western and already highly religious societies.

To address this gap, we used a nationally representative sample from Saudi Arabia, a predominantly religious and conservative society where Islam is the major faith. The present study confirmed that religiosity was associated with less mental health disorders. It is important to note that although the association weakened after adjusting for other variables, the 95% confidence intervals (*OR*: 0.95, 95% *CI*: 0.90–1.00, *p* = 0.067) suggests that most of the plausible effect range favors a positive role.

Previous studies on this topic often did not fully distinguish between religiosity and spirituality. Religiosity generally refers to adherence to an organized system of beliefs, practices, rituals, and symbols designed to facilitate closeness to God or other deities. Spirituality, on the other hand, refers to a personal search for ultimate meaning in life and for a relationship with the “sacred,” which may or may not be tied to a formal religion (38). While the two constructs overlap, they are regarded as distinct. Dubey et al. (39) reviewed the literature to quantify the distinct effects of religiosity and spirituality on psychological well-being and their review highlighted the need for more studies that separate these dimensions, as they are often conflated in the literature.

As such, our study focused exclusively on religiosity, using a comprehensive measure that captured multiple dimensions of the Islamic faith. It included belief, morals, worship, and other prescribed practices in order to generate an overall score of religiosity for each respondent. The findings indicated that religiosity alone, even without a strong spirituality component, is generally positively associated with mental health. Future research should examine spirituality independently to clarify its relationship with mental health outcomes. It is plausible that the combination of both religiosity and spirituality provides the strongest positive effect on mental health. This may also explain why our study's results showed slightly overlapping confidence intervals, suggesting some imprecision in the independent effects of religiosity.

Many published studies have suggested that religion may function as an effective coping mechanism for chronic pain and fatigue (40, 41), as well as for headaches such as migraines (42, 43) demonstrating positive effects on well-being. Notably, one randomized trial compared treatment of migraine with 40 mg of medication alone vs. 40 mg of medication combined with prayer. The group receiving medication plus prayer reported significantly lower pain intensity at 3-month follow-up (44). Given these results, one might expect religiosity to modify the relationship between headaches and mental health. However, the present study did not find evidence for such a modifying effect and the association appeared similar across different religiosity levels.

One plausible explanation for the lack of a significant interaction is the limited heterogeneity in religiosity within the Saudi sample. In our study, 92% of respondents were classified as highly religious, while only 8% were moderately religious. Although a composite religiosity score was used in the model, the limited variation may have limited our ability to detect a true interaction effect. Replicating this analysis in populations with greater variability in religiosity could provide more definitive answers.

Despite the lack of significant interaction, we believe that these findings indicate the persistence of headache-mental disorder link across all levels of religiosity. In other words, strong faith alone did not eliminate the mental health issues for those with headaches. It underscores the importance of screening and seeking professional help regardless of religiosity levels. Our previous studies using the SNMHS data have shown that a large proportion of the Saudi population does not seek mental health treatment, often due to stigma and lack of awareness, and many instead turn to religious advisors (45, 46). This pattern is not unique to Saudi Arabia; Khalifa et al. (47) observed similar findings in the Muslim population in the United Kingdom, where individuals cited religious figures more often than medical doctors for mental health concerns. Religious advisors are not formally trained in mental health counseling and often lack access to appropriate resources. Given the central role of religion in the lives of many Muslims and religious individuals, we advocate for a collaborative model that integrates mental health professionals with religious advisors. Such an approach may offer a promising strategy to improve access to care in highly religious societies like Saudi Arabia. This collaborative approach aligns with Islamic tradition, which encourages reliance on God (*Tawakkul*) while also seeking professional help, as exemplified in the *Sunnah* (the practices of the Prophet Muhammad, peace and blessings be upon him). Similar strategies are being explored in the United Kingdom (48, 49) and could be adapted to other contexts.

There are several limitations to this study. First, the SNMHS is a cross-sectional survey, and therefore causal inferences cannot be drawn. It remains unclear whether higher levels of religiosity lead to fewer mental health issues, or whether individuals with fewer mental health issues are more likely to engage in religious practices. Similarly, the relationship between headaches and mental health is likely bidirectional, with each potentially influencing the other; elucidating the precise mechanisms was beyond the scope of this study. Second, headache was operationalized using a broad self-reported question rather than formal diagnostic criteria. Although this approach is appropriate for an epidemiological cross-sectional study such as ours, future studies should extend this work by evaluating headache prevalence using physician-diagnosed cases or standardized diagnostic criteria. Third, although the present study used nationally representative data and adjusted for several key confounders, residual confounding remains possible, particularly from variables that were not measured or collected, such as sleep disturbances and other sources of stress exposure. Fourth, as with all surveys, systematic non-reporting is a concern. Respondents may be reluctant to disclose information on religiosity or mental health. However, trained interviewers emphasized confidentiality and privacy to minimize reporting bias. Finally, mental health diagnoses were made using the WHO CIDI, rather than clinician-administered interviews, which may have resulted in some overestimation of prevalence. Nevertheless, many of our findings are consistent with studies in other countries and cultural contexts, suggesting that these limitations are unlikely to substantially alter the main conclusions.

## Conclusion

Headaches are common among Saudis and are associated with mental health disorders. However, varying levels of religiosity do not appear to modify this relationship. These findings underscore the importance of screening for mental health disorders in individuals presenting with headaches, regardless of their religiosity, and highlight the potential value of collaborative models that integrate professional mental health support with religiously sensitive approaches.

## Data Availability

The datasets presented in this article are not readily available because of restrictions in the informed consent language used to recruit respondents and WMH consortium agreements. Requests to access the datasets should be directed to the corresponding author at sdesai@kfshrc.edu.sa.
