# Supplementary material for: Headaches, mental health disorders, and religiosity: analysis of the Saudi National Mental Health Survey
Source: Front Public Health. 2026 Apr 15;14:1813677. doi: 10.3389/fpubh.2026.1813677 (PMC13124478; doi:10.3389/fpubh.2026.1813677)
Supplement: Supplementary file 1 [file Table_1.docx]

**Supplementary material**

**Figure S1: Contents of the Islamic Religiosity Scale in English and Arabic**

**
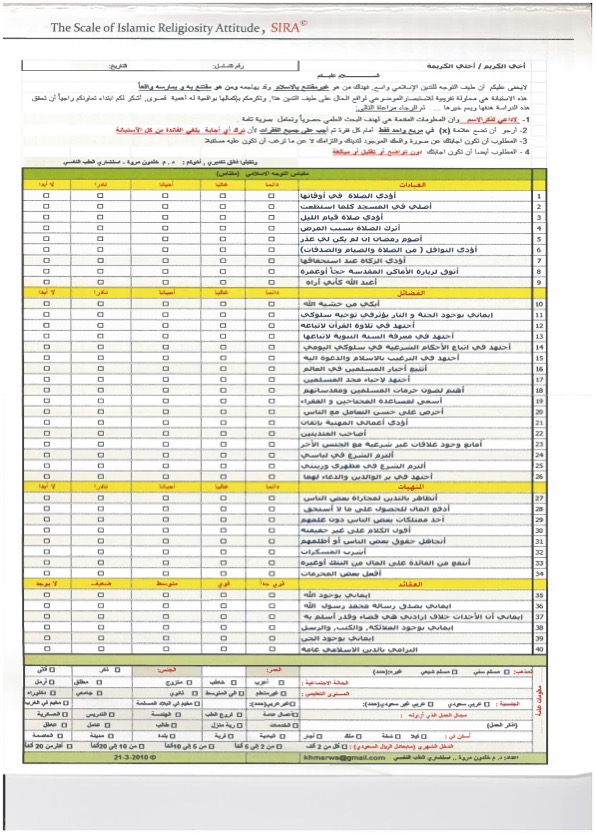
**

**Table S1: Relationship between different mental health disorders and headaches**

| **MH disorder** | **OR**^1^ | **95% CI**^1^ | **p-value** |
| --- | --- | --- | --- |
| DSM_PDS |  |  |  |
|  | — | — |  |
|  | 1.45 | 0.72, 2.92 | 0.3 |
| **DSM_AGO** |  |  |  |
|  | **—** | **—** |  |
|  | **2.88** | **1.26, 6.59** | **0.012** |
| **DSM_SO** |  |  |  |
|  | **—** | **—** |  |
|  | **2.83** | **1.64, 4.90** | **<0.001** |
| DSM_GADH |  |  |  |
|  | — | — |  |
|  | 1.80 | 0.67, 4.86 | 0.2 |
| **DSM_PTS** |  |  |  |
|  | **—** | **—** |  |
|  | **3.51** | **2.01, 6.14** | **<0.001** |
| **DSM_ASA_SAD** |  |  |  |
|  | **—** | **—** |  |
|  | **1.98** | **1.20, 3.27** | **0.008** |
| **DSM_OCD** |  |  |  |
|  | **—** | **—** |  |
|  | **2.84** | **1.43, 5.64** | **0.003** |
| **DSM_MDDH** |  |  |  |
|  | **—** | **—** |  |
|  | **2.34** | **1.42, 3.85** | **<0.001** |
| DSM_BIPOLAR |  |  |  |
|  | — | — |  |
|  | 2.50 | 0.97, 6.42 | 0.057 |
| **MOOD_LT** |  |  |  |
|  | **—** | **—** |  |
|  | **2.48** | **1.55, 3.98** | **<0.001** |
| DSM_ADD |  |  |  |
|  | — | — |  |
|  | 1.14 | 0.63, 2.04 | 0.7 |
| **DSM_CD** |  |  |  |
|  | **—** | **—** |  |
|  | **3.43** | **1.59, 7.38** | **0.002** |
| DSM_IEDH |  |  |  |
|  | — | — |  |
|  | 1.16 | 0.57, 2.34 | 0.7 |
| IMPULSE_LT |  |  |  |
|  | — | — |  |
|  | 1.34 | 0.82, 2.19 | 0.2 |
| DSM_ALAH |  |  |  |
|  | — | — |  |
|  | 5.87 | 0.67, 51.2 | 0.11 |
| DSM_ALD |  |  |  |
|  | — | — |  |
|  | 0.71 | 0.13, 3.79 | 0.7 |
| DSM_DRAH |  |  |  |
|  | — | — |  |
|  | 0.76 | 0.34, 1.70 | 0.5 |
| DSM_DRD |  |  |  |
|  | — | — |  |
|  | 2.46 | 0.84, 7.19 | 0.10 |
| SUBSTANCE_LT |  |  |  |
|  | — | — |  |
|  | 0.94 | 0.49, 1.82 | 0.9 |
| DSM_ANO |  |  |  |
|  | — | — |  |
|  | 0.11 | 0.01, 1.23 | 0.074 |
| DSM_BUL |  |  |  |
|  | — | — |  |
|  | 0.79 | 0.38, 1.64 | 0.5 |
| **DSM_BINGEH** |  |  |  |
|  | **—** | **—** |  |
|  | **2.14** | **1.07, 4.26** | **0.032** |
| EATING_LT |  |  |  |
|  | — | — |  |
|  | 1.05 | 0.56, 1.96 | 0.9 |

**Table S2: Relationship between life-time prevalence of mental health disorders and headaches by Gender**

|  | **OR**^1^ | **95% CI**^1^ | **p-value** |
| --- | --- | --- | --- |
| **headache_life** |  |  |  |
| No | — | — |  |
| Yes | 2.17 | 1.34, 3.53 | 0.002 |
| **sex_cat** |  |  |  |
| Male | — | — |  |
| Female | 1.10 | 0.64, 1.88 | 0.7 |
| **headache_life * sex_cat** |  |  |  |
| Yes * Female | 0.80 | 0.41, 1.55 | 0.5 |
| ^1^OR = Odds Ratio, CI = Confidence Interval | | | |

**Table S3: Relationship between Religiosity score (as continuous term) and life-time mental health disorder**

|  | **OR**^1^ | **95% CI**^1^ | **p-value** |
| --- | --- | --- | --- |
| **Religiosity level score (per 5-point increase)** | 1.03 | 0.98, 1.09 | 0.20 |
| ^1^OR = Odds Ratio, CI = Confidence Interval | | | |

**Table S4: Relationship between Religiosity score (using b splines) and life-time mental health disorder**

**
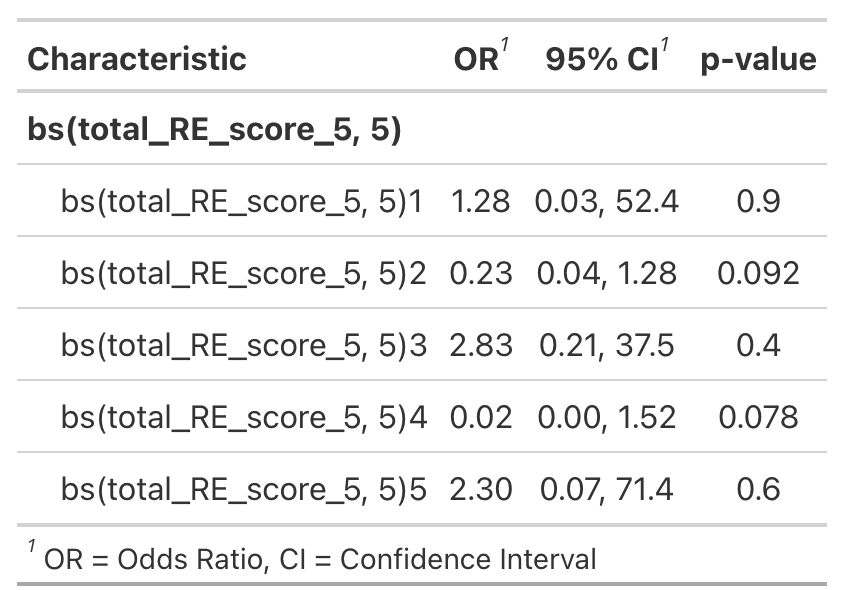
**

ANOVA test for comparing linear model (Table S4) to non-linear model (Table S5):

Working 2logLR = 7.425309 **p= 0.1252**

(scale factors: 1.7 1.3 0.54 0.44 ); denominator df= 1869

**Table S5: Relationship between Religiosity score (using categories) and life-time mental health disorder**

**
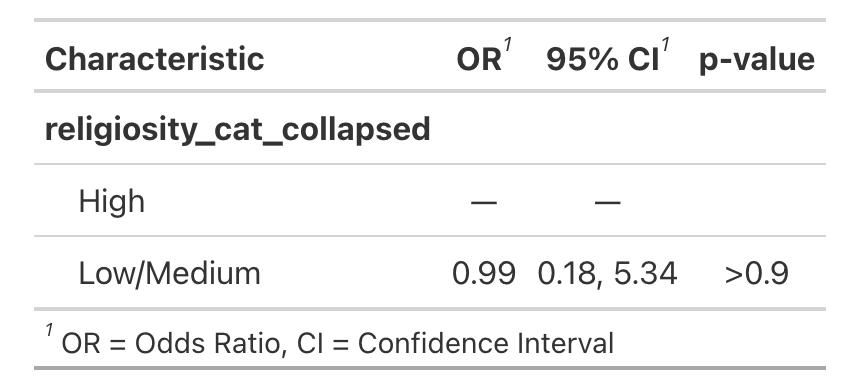
**

**AIC for Table S6 (categorical model) = 2421**

**AIC for Table S4 (linear model) = 2420 (lower AIC is better)**

**Table S6: Predicted probabilities of having a life-time mental health disorder and 95% CIs for different levels of religiosity score (higher scores means greater religiosity) and having headaches**

| **Religiosity score** | **Predicted probability of mental health disorders (95% CI)** | **Difference in probability between headaches and no headaches** |
| --- | --- | --- |
| **166 (first quartile)** | | |
| Headaches = No | 29% (23%, 36%) | +16% |
| Headaches = Yes | 45% (39%, 50%) |  |
| **175 (mean)** |  |  |
| Headaches = No | 26% (21%, 32%) | +16% |
| Headaches = Yes | 42% (37%, 46%) |  |
| **188 (third quartile)** |  |  |
| Headaches = No | 23% (17%, 29%) | +14% |
| Headaches = Yes | 37% (31%, 43%) |  |

**Figure S2: Plot of Predicted probabilities of having a life-time mental health disorder and 95% CIs for different levels of religiosity score (higher scores means greater religiosity) and having headaches**

**
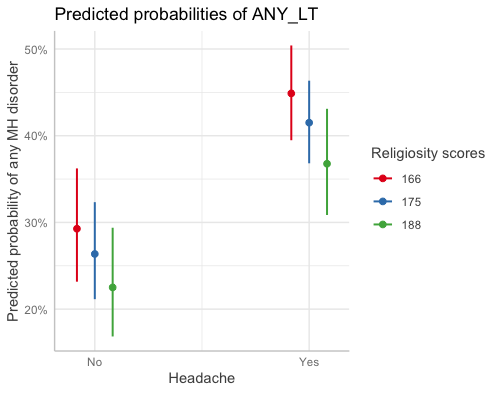
**
